# Could COVID-19 improve psychiatric awareness at the heart of the Middle East? – A personal reflection on Bahrain’s response

**DOI:** 10.1017/ipm.2020.74

**Published:** 2020-06-02

**Authors:** H. M. Negm

**Affiliations:** Child & Adolescent Liaison Psychiatry, Children’s Health Ireland at Crumlin, Dublin, Ireland

**Keywords:** Bahrain, coronavirus, COVID-19, mental health

## Abstract

This perspective offers a personal insight into COVID-19 in Bahrain along with the response to this unprecented pandemic. In a country where a robust health care system and economic prosperity have allowed it to cope with the medical sequelae, the mental health consequences may have been less anticipated but more problematic. An unforeseen positive emerging from the pandemic might be the nation’s recognition of the importance of mental health well-being and a new openness to discussing it.

The Gulf states have not been immune to the unprecedented public health challenges which have occurred secondary to the COVID-19 pandemic. The fight against COVID-19 has steered oil-rich countries to a new way of life and self-perception. The unexpected surge of the disease in bordering countries restricted time available to introduce measures to tackle COVID-19. One of these surges was the Iranian COVID-19 outbreak which could be considered as the Middle East’s epicentre in the devastation inflicted by the disease. The rapid and unpredictable mayhem that followed took the small kingdom of Bahrain by surprise.

The island is known for its pearl trade heritage, the relaxed nature of its people and its fragrant desserts. But the country is also known as a historical pivot point in the gulf region with a civilisation considered to be as old as 5,000 years. This gives it a unique demographic distribution, with a majority Shiite Muslim population with strong societal, religious and cultural links to the Republic of Iran. Concern regarding the high number of cases in Iran coincided with lockdown challenges at the time of Nowruz, the New Year celebrations. By 23 February, cases of Iranian origin had surfaced in Canada, Lebanon and the United Arab Emirates. Given the country’s proximity to Iran, coupled with large numbers of Bahraini pilgrims in Iranian cities, it was only a matter of time before the small archipelago faced its own battle with COVID-19.

The World Health Organization (WHO) declared the COVID-19 outbreak a Public Health Emergency of International Concern on January 30 2020; on March 11, it was reclassified as a pandemic. On February 19, it was reported that two people tested positive for COVID-19 and subsequently died in the city of Qom, Iran. This sent alarm across the region, as the ancient city is considered a holy site in Shi’a Islam and is visited by many Shiite Muslims from all over the world. On February 24 2020, Bahrain reported its first confirmed case of COVID-19, a school bus driver who had travelled back from Iran. In the course of 24 hours, the Bahraini Ministry of Health announced a total of 23 confirmed cases of COVID-19 in the country, with the overwhelming majority having travelled either directly or indirectly from Iran. The Bahraini Civil Aviation Affairs immediately responded by suspending all flights from the United Arab Emirates and Iranian Airports for a 48-hour period, which subsequently extended into a complete shutdown, leaving many Bahraini pilgrims stranded in Iran. By April 19 2020, there were 1,773 confirmed cases with 7 deaths in Bahrain; at the time of writing there is no indication that these numbers are slowing down.

As the crisis created havoc, voices of displeasure emerged from Bahraini citizens as they tried to adjust to the strict 14-day quarantine and compulsory heath checks post-travel, many of whom were Shiite pilgrims visiting Iran. A sentiment of stigmatisation and victimisation towards Shiite pilgrims could somehow be sensed on social media, reawakening previously held views that a sectarian divide contributed to economic disparity and the denial of civil rights (The Guardian, 2011).

Despite the majority of Bahrainis (about 70%) being Shia, the majority of the government, military and business leaders are Sunni. When authorities made a decision to keep nearly a thousand Bahraini citizens in Iran after lockdown, old wounds may have re-opened. The hashtag ‘#Return_Bahrainis_Stuck_Outside’ was a source of debate and controversy in the Bahraini political arena.

On a different note, the financial impact of COVID-19 on other Gulf states in general has been substantial. With the price of oil plummeting to unprecedented levels, there seems to be a struggle to maintain aneconomic balance and, coupled with the extinction of tourism and the postponement of the Bahrain Formula 1 Grand Prix, economic hardship has now been added to an already tense environment nationally with demands on the government to respond.

Bahrain’s small size and small population, along with a universal health care system and well-developed infrastructure, helped in the fight against the contagion. Bahrain was one of the very few Middle Eastern countries to start a national information campaign on COVID-19, outlining early signs and symptoms as well as methods of prevention and recommendations to the public on management of the disease. From as early as February, the country started to employ vigorous efforts in isolating cases at all points of entry. Bahrain followed WHO guidelines on measures of social distancing resulting in the closure of busy malls, markets, bars, cafes, shisha places and mosques. A complete lockdown ensued to include parks and causeways by mid-February. Curfews were introduced from 6pm to 5am and electronic wrist bands monitored home-quarantined individuals with strict penalties for non-compliance. April heralded the additional mandate of requiring all citizens to wear masks in public areas.

In the health sector, isolation and quarantine field triaging facilities were developed in hotels, initially at the expense of individuals, but later free of charge; carparks and mobile clinics were also established on public buses. Efforts were made to isolate cases early and to prevent further exposure to patients and healthcare staff with new COVID-19-specific operating theatres established for urgent surgeries. Tele-consultations for all outpatients were set up from March for low-risk patients, while those deemed high-risk or in need of urgent medical care undergoing a triaging process. Such a broad swathe of innovation reflected the kingdom’s unprecedented healthcare efforts, matching those of other developed countries.

The psychological impact of the pandemic on the country’s people and economy has slowly begun to percolate through Bahrain. The Middle East has a relatively young and, as yet, under-developed mental health system in terms of infrastructure and financial commitment. Although Bahrain fares better than many other Arab countries in terms of mental health dedicated hospital beds and psychiatry personnel, numbers are far below requirements (Okasha *et al.* 2012). Cultural beliefs regarding the origins and treatments of mental illness differ, with some wealthy families choosing to seek treatment abroad (Al-Ansari *et al*. 2013), while others seek support from traditional healers (Okasha *et al*. 2012). Even when families seek help from the mental health services, they experience more stigmatising attitudes than patients attending other medical/surgical services (Al Saif *et al*. 2019). This may operate as a major barrier to help-seeking behaviour for citizens experiencing a deterioration in mental health or new onset mental health symptoms at the time of COVID-19.

Although psychiatry services in Bahrain were slow to develop – the first drug approved for treatment of mental illness came in 1964 and community mental health services were established in 1979 (Al-Haddad & Al-Offi, 2009) – improvements have been swift. Decentralisation has resulted in psychiatric services being provided at district hospitals and local units, making them more accessible. Training in mental health has expanded to include general practitioners and health personnel working at a primary health care level (Okasha *et al*. 2012). The expected increase in mental health presentations in the wake of COVID-19 will test both the attitudes and service capability of providers.

As of May 12, cases infected with COVID-19 stood at 5,816 with 10 deaths. However, the psychological ramifications of this illness will be severe. As mental health professionals, we are facing a new cohort of psychiatric presentations compounded by the global pandemic, along with the profound societal implications of the crisis. The social and financial deprivations impacting many sectors of society, the at times panic-inducing mass media coverage, and the deprivation of movement has led to ubiquitously heightened stress levels. But it has also triggered a set of questions that were in the past rarely asked in this part of the world: ‘Are you okay?’; ‘Are you sad?’; ‘How are you coping?’ and ‘Do you feel lonely?’ Such questions are emerging and are aided by global communication networks that are essentially sympathetic to the cause of ‘How to cope’. In countries such as Bahrain where the awareness of mental health issues and the willingness to talk about them has been limited historically, these questions are welcome.

Tele-consultations are now standard, facilitating follow-up with psychiatric patients by mental health professionals. A mobile application called ‘Doctori’ (Arabic for ‘My Doctor’) was another hopeful sign that psychiatry is being given due recognition. The creators of the application further expanded its use for psychotherapy and, later, to other specialties of medicine with further plans to expand to other Gulf states. Several hospitals in Bahrain have initiated tele-conference symposiums set-up by Psychiatrists and Psychologists to talk to medical health workers about strategies to cope with the impact of COVID-19 on their own mental health while caring for patients in need. Furthermore, news of designated hotlines for medical practitioners to speak about their psychological struggles amidst the crisis were established.

Before the COVID-19 crisis, improvements were being made in the mental health services in Bahrain and in the promotion of mental health and well-being, but it was recognised that more was needed especially if preventative measures are to be established (Osaka *et al*. 2012). The Bahraini government has recently injected an $11 billion stimulus package to aid the private sector to cope with the repercussions of COVID-19, but how much will be directed to mental health has yet to be seen. Hopefully, the COVID-19 pandemic will draw attention to the need for emotional and psychological solidarity to overcome this global threat, as well as increasing awareness of the fundamental importance of mental health for survival.
